# Scenes Modulate Object Processing Before Interacting With Memory Templates

**DOI:** 10.1177/0956797619869905

**Published:** 2019-09-16

**Authors:** Surya Gayet, Marius V. Peelen

**Affiliations:** Donders Institute for Brain, Cognition and Behaviour, Radboud University

**Keywords:** visual search, visual attention, visual memory, visual perception, scene perception, attentional capture, open data, open materials

## Abstract

When searching for relevant objects in our environment (say, an apple), we create a memory template (a red sphere), which causes our visual system to favor template-matching visual input (applelike objects) at the expense of template-mismatching visual input (e.g., leaves). Although this principle seems straightforward in a lab setting, it poses a problem in naturalistic viewing: Two objects that have the same size on the retina will differ in real-world size if one is nearby and the other is far away. Using the Ponzo illusion to manipulate perceived size while keeping retinal size constant, we demonstrated across 71 participants that visual objects attract attention when their perceived size matches a memory template, compared with mismatching objects that have the same size on the retina. This shows that memory templates affect visual selection after object representations are modulated by scene context, thus providing a working mechanism for template-based search in naturalistic vision.

The ability to rapidly detect behaviorally relevant objects within a rich visual environment provides a clear adaptive advantage. To agents navigating through a complex and dynamic world, the currently relevant object might differ from the objects that were relevant a day ago or even a few seconds ago. To account for this, nature has equipped human observers with the ability to strategically filter the influx of retinal input in a goal-directed manner (e.g., Desimone & Duncan, 1995). Extant theories of visual search described this process as follows: When searching for a relevant item (say, an apple) in our visual environment, we maintain a visual template in memory (e.g., a representation of a small circular red object), which causes our visual system to favor template-matching visual input (e.g., apples) at the expense of template-mismatching visual input (e.g., leaves of the apple tree). This fundamental principle of human vision underlies all major theories of visual search (Desimone & Duncan, 1995; Duncan & Humphreys, 1989; Eimer, 2014; Kastner & Ungerleider, 2001; Wolfe, 1994; Wolfe & Horowitz, 2004).

Although template-based visual selection has been extensively studied in laboratory settings, where objects are presented in isolation, it remains an open question whether this principle generalizes to naturalistic vision outside of the laboratory (Wolfe & Horowitz, 2004; Wolfe, Võ, Evans, & Greene, 2011), where objects are presented in context (Bar, 2004; Oliva & Torralba, 2007). One key property of naturalistic vision constitutes a particular challenge for template-based visual selection: The image that an object produces on the retina depends on where the object is situated in the real world. For instance, the light source, viewpoint, and distance of an object in the real world dramatically alter the brightness, color, shape, and size of its image on the retina. Consequently, the visual system first needs to account for the context in which an object is situated before a concurrent memory template can favor this object over irrelevant visual input. This would entail, for example, first rescaling the representation of an object to account for viewing distance and then comparing the rescaled representation with a canonically sized memory template. Alternatively, if this were not the case, observers would need to continuously adjust their memory template to match the retinal image that an object would produce at a given location (e.g., generating smaller templates to search at greater distance). To the best of our knowledge, it remains unknown whether memory templates impact the visual-processing stream before or after object representations are modulated by their visual context. Consequently, it remains unknown how template-based visual selection is applicable to naturalistic viewing.

The critical role of context in naturalistic viewing is arguably best exemplified by differences in distance between an object of interest and the observer: Two objects that produce an image of the same size on the retina can be of vastly different sizes in the real world if one is nearby and the other one is farther away. Human observers rarely mistake a small, nearby object (say, a toy car at a distance of 1 m) for a large object at greater distance (an actual car at a distance of 20 m), despite significant overlap in visual characteristics. In fact, observers will often fail to detect an object when it produces a retinal image of a size that is incompatible with the object’s canonical size at the inferred distance from the observer (Eckstein, Koehler, Welbourne, & Akbas, 2017). Thus, observers utilize the inferred distance to an object of interest to derive an estimate of the size that this object should produce on the retina. In addition, differences in inferred distance also strongly affect the perceived size of an object (e.g., Gregory, 1968).

In the current study, we capitalized on a variant of the Ponzo illusion (Ponzo, 1911) to manipulate the perceived size of a visual object by altering its context. Specifically, we made objects of fixed retinal size appear larger or smaller by positioning them at locations that corresponded to either the near plane or the far plane of a naturalistic scene. In order to manipulate participants’ memory template, we asked participants to concurrently maintain either a smaller or larger version of the object in memory for later recall. This allowed us to investigate whether visual objects that perceptually match the size of a memory template are favored over mismatching visual objects, even when the competing objects have the same size on the retina.

A visual-probe paradigm was used to assess whether memory templates cause the visual system to systematically favor template-matching over template-mismatching visual objects. In this paradigm, two competing images are briefly presented (in this case, a template-matching and a template-mismatching visual object) and immediately followed by an unrelated target presented at the location of one of the two competing images. Better target detection or discrimination performance at the location of one of the two images provides evidence that this image was favored by the visual system (i.e., it captured attention) relative to the competing image (for a similar approach, see Jiang, Costello, Fang, Huang, & He, 2006; Reeder & Peelen, 2013). In our case, we hypothesized that participants would be faster at reporting the orientation of a target grating if it appeared at the location of the template-matching object (i.e., a distant object when a large item was memorized or a near object when a small item was memorized) than at the location of the template-mismatching object. This would indicate that the visual system favored visual objects whose perceived size, as inferred from the context, matched the current memory template.

In Experiment 1, we demonstrated that template-matching visual objects are favored over template-mismatching objects, even when the competing objects produce the same retinal image. This effect was replicated in Experiments 2 and 3, following a power analysis based on the data of Experiment 1. Additionally, Experiment 2 demonstrated that this effect genuinely relies on the perception of depth induced by the scenes, as the effect was not observed with control scenes that did not induce a perception of depth. Moreover, the effect correlated with the degree to which individual scenes induced a size illusion for a given participant. Finally, Experiment 3 confirmed that attentional resources are allocated to template-matching objects automatically (i.e., through involuntary capture of attention) rather than strategically.

## General Method

### Participants

Participants were gathered via the Radboud University online recruitment system (Sona Systems) and were compensated with course credit or monetary reward. All participants had normal or corrected-to-normal vision, were no older than 30 years of age, and provided written informed consent prior to participation. The study was approved by the Faculty of Social Sciences Ethics Committee (ECSW2017-2306-517).

In Experiment 1, we collected data until 20 participants met our inclusion criteria (2 participants were replaced, following the exclusion criteria described in the Data Selection and Preparation section). Because of the exploratory nature of this first experiment, the sample size was based on data from earlier studies in which the influence of color (rather than size) templates on attentional capture was investigated (e.g., Olivers, Meijer, & Theeuwes, 2006; van Moorselaar, Theeuwes, & Olivers, 2014). The final participant group had an average age of 24.2 years (*SD* = 3.7), and 8 of the participants were male.

For Experiment 2, the sample size was determined through a power analysis based on the data of Experiment 1. This analysis revealed that a sample size of 25 participants was required to obtain 80% power to detect a difference in response times (RTs) between template-matching trials and template-mismatching trials at least as large as the one observed in Experiment 1, on the basis of a simple *t* contrast. Thus, data acquisition in Experiment 2 continued until 26 participants met our inclusion criteria, 13 in each counterbalancing condition (5 participants were replaced). The final pool of participants had an average age of 21.7 years (*SD* = 2.8), and 6 of the participants were male.

On the basis of the same power analysis, we continued data acquisition for Experiment 3 until 25 participants met our inclusion criteria (3 participants were replaced). Nine of the participants in the final pool of participants were male, and their average age was 23.2 years (*SD* = 3.6).

### Procedure

#### Experiment 1

Experiment 1 consisted of two parts. The first part (the *main experiment*) was designed to investigate whether template-matching visual objects are favored over template-mismatching visual objects. Before participating in four blocks of 32 trials each (128 trials in total, or 64 per condition of interest), participants viewed a step-by-step demonstration of the trial sequence and performed 20 practice trials.

**Fig. 1.**
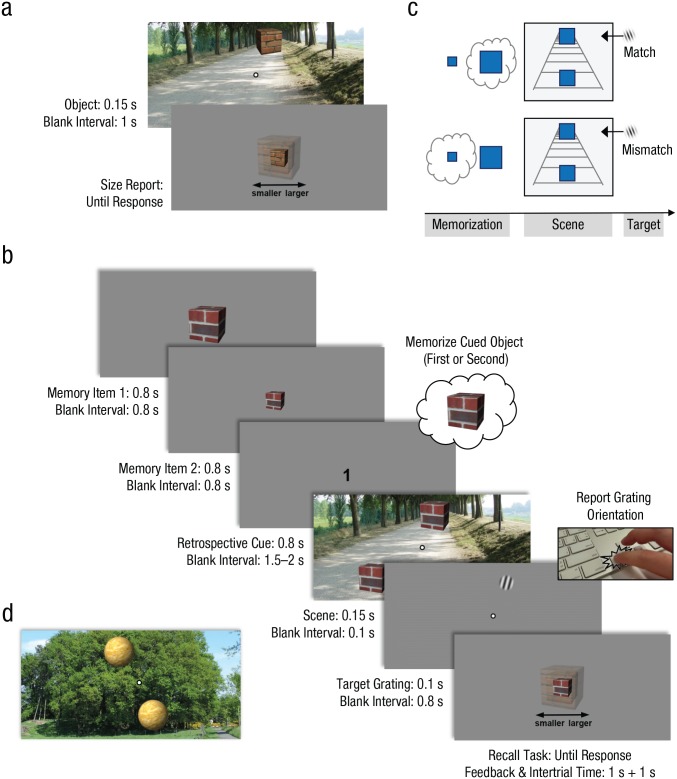
Example trial sequence and stimuli for Experiments 1 and 2. On each trial of the size-illusion measurement (a), an object was presented either nearby or far away in a scene, after which participants were asked to reproduce the size of the object by up- or downscaling a test object. In the main experiment (b), participants were retrospectively cued to memorize the exact size of the first or second object they had seen for subsequent recall. During the retention interval, participants performed a speeded orientation-discrimination task on a target grating that appeared at a location that was preceded by either a template-matching visual object (e.g., a perceptually large object when a large object had been memorized; shown here) or a template-mismatching visual object (e.g., a perceptually large object when a small object had been memorized; not shown). The same visual stimulation led to matching and mismatching trials (c): Depending on whether the large or small object was memorized, either the nearby or the distant object matched the template (and gratings presented at that object’s location were hypothesized to yield faster response times). In Experiment 2, a condition was included with scenes that should not induce a size illusion (no-depth control condition; d provides one example).

Each trial (illustrated in Fig. 1b) started with a 1-s fixation interval. Next, a relatively large visual object was presented at fixation, followed by a relatively small visual object (or vice versa) and then a retrospective cue indicating which of the two object sizes should be memorized for later recall (a “1” or “2” instructed participants to memorize the first or second object, respectively). During the retention period, a scene comprising two intermediate-sized versions of the same object was presented for 150 ms (1.5 s to 2 s after the cue). One of these objects was presented above fixation (corresponding to a “distant” location), and one was presented below fixation (corresponding to a “nearby” location), at a vertical distance of 2.1° of visual angle. Importantly, the distant object would appear larger than the nearby object, despite being identical in retinal size. Participants were instructed that these task-irrelevant scenes could be ignored but that they would be followed shortly by a task-relevant target. After 100 ms, a small target grating was briefly presented (at 0.8° of visual angle; 100 ms) at the same location as one of the two previously presented objects. The grating was tilted 10° clockwise or counterclockwise from the vertical midline, and participants were instructed to report its tilt as quickly and accurately as possible. After they provided their response, participants were presented with a randomly sized variant of the memorized object, which they were required to adjust until it matched the exact size of the memorized object. This task was not speeded, and participants received feedback on their accuracy after each response: a green (< 15% error), orange (< 28.5% error), or red (> 28.5% error) outline of the correct size was displayed on top of the reported size. At the end of each block, participants received feedback on their average accuracy on the memory-recall task, as well as on their average RT and accuracy on the orientation-discrimination task.

The second part (the *size-illusion measurement*) was designed to assess whether our stimuli successfully elicited a size illusion, that is, visual objects presented far away appeared larger than the same visual objects presented nearby. Participants performed two blocks of 32 trials, or 64 trials in total (32 per distance condition). Each trial (illustrated in Fig. 1a) started with a 1-s fixation interval. Next, 1 of 16 possible scenes was briefly presented with either a near or a distant object. After a 1-s delay, that same object (but of a random size) was displayed in isolation at fixation, and participants were asked to rescale it until it matched the size (in pixels) of the visual object that had just been presented in the scene. No feedback was provided. All stimulus properties (e.g., timing, stimulus sizes) were identical to those in the main experiment.

#### Experiment 2

Experiment 2 was identical to Experiment 1, except that participants now participated in two experimental sessions on separate days (the order of which was counterbalanced across participants). One of these sessions was a direct replica of Experiment 1, and in the other session, the depth-inducing scenes were replaced with “flat” control scenes, which we expected would not differentially affect the perceived size of the top and bottom objects (Fig. 1d).

#### Experiment 3

**Fig. 2.**
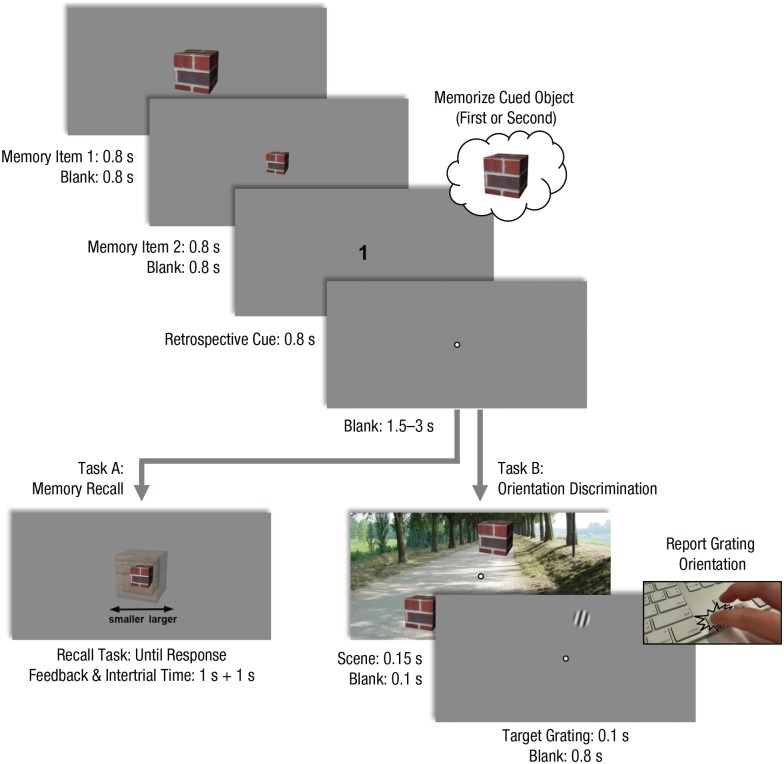
Example trial sequence in Experiment 3. Trials in Experiment 3 were identical to those in Experiments 1 and 2, with one crucial distinction: On each trial, after participants were cued to memorize the size of the large or small object, they performed either the memory task or the orientation-discrimination task but never both. The two trial types were intermixed. This adaptation allowed us to ensure that participants would not strategically allocate more attentional resources to one of the objects in the scene in the belief that this would help them for the ensuing memory-recall task.

The main experiment of Experiment 3 was identical to that of Experiment 1, except that participants now performed either the memory-recall task or the orientation-discrimination task on any given trial but never both (see Fig. 2). Critically, the two trial types were intermixed to incite participants to memorize the retrospectively cued object on each trial. Finally, there was no size-illusion measurement in Experiment 3.

### Stimuli

Sixteen different naturalistic scenes were retrieved via Google image search and cropped to a height of 6.3° of visual angle and a width of 14°. These scenes were images of roads, paths, runways, or train tracks, which were selected (and pilot tested) to induce a sense of depth (Fig. 1a). The upper part of the image depicted a location that was farther away from the viewer than the lower part of the image. Experiment 2 included 16 additional natural scenes, also retrieved from Google image search, for a separate control condition. These scenes featured walls, hedges, buildings, cliffs, and similar images, and the upper part of the image did not depict a location farther away from the viewer than the lower part of the image (Fig. 1d).

The visual objects presented in the scenes, and presented in isolation for the memory task, consisted of 40 different visual objects created in the GNU Image Manipulation Program (https://www.gimp.org)—either cubes or spheres, with 1 of 20 different textures (see Figs. 1a, 1b, and 1d for different examples of objects).

To keep the memory task challenging, we varied the sizes of the visual objects throughout the experiment. The eventual object sizes were obtained by multiplying the native size of the visual object (0.7° × 0.7° of visual angle) by predetermined factors. First, on each trial, 1 of 20 possible base object sizes was drawn, ranging from 15% smaller to 15% larger than the native object. The competing visual objects within the scenes were always of this base object size. To obtain the small and large objects for the memory task, we decreased and increased the base object size by 28.5%; in addition, we applied 1 of 10 possible size variations ranging from 8% smaller to 8% larger than the resulting size. The same size variation was applied to the cued (i.e., to be memorized) and the noncued (i.e., to be discarded) objects of the memory task.

Participants provided a speeded report of the orientation of a target stimulus. This target stimulus was a gray-scale sine-wave grating (with the same mean luminance as the gray background), which was rotated 10° clockwise or 10° counterclockwise from the vertical midline. More details on the stimuli and the experimental setup are provided in the Supplemental Material available online (see Section S.5).

### Experimental design

In the main experiment, there was one dependent variable (RT to the target grating) and one factor of interest: congruence (i.e., whether the target grating appeared at the location of the template-matching or template-mismatching visual object; Fig. 1c). Three additional factors were also fully counterbalanced within each of the four experimental blocks: template size (participants memorized either the large or the small object), retrospective cue (participants memorized either the first or the second object), and grating orientation (the target grating was tilted 10° clockwise or counterclockwise from the vertical midline). Each specific combination of these counterbalanced conditions was repeated twice within each block and presented in randomized order. In addition, a number of factors were not counterbalanced, but their prevalence was optimally equated between blocks, and they were presented in random order. This included the 16 different scenes, two different object shapes (cube or sphere), 20 different object textures, 20 different base object sizes, 20 different size variations for the to-be-memorized objects, 10 horizontal positions for the objects in the scene, and 32 different initial sizes for the test object in the recall phase.

In the size-illusion measurement, there was also one dependent variable (reported object size) and one factor of interest: distance (whether the visual object was distant or nearby). Two additional factors were also fully counterbalanced: the scene (16 variations) and the object shape (cube or sphere). Each specific combination of these counterbalanced conditions was repeated once within the entire 64-trial size-illusion measurement, and the combinations were presented in randomized order. A number of additional factors were not counterbalanced, but their prevalence was optimally equated: 20 different object textures, 16 different object sizes, 10 horizontal positions for the objects in the scene, and 32 different initial sizes for the test object in the recall phase. (Table S.1 in the Supplemental Material provides an overview of all experimental factors in our design.)

### Data selection and preparation

Participants were excluded from further analysis if they performed at chance on either the orientation-report task (i.e., not better than 50% correct, as determined with a one-sided *t* test) or on the memory-recall task (i.e., size error not below 28.5%, as determined with a one-sided *t* test) of the main experiment. The threshold of 28.5% reflects the minimally required recall precision for distinguishing between the sizes of the cued (i.e., to-be-memorized) and uncued (i.e., to-be-discarded) objects in the memory task, whose size differed by 57%. Errors beyond 28.5% thus reflect a category error (i.e., the wrong size category was memorized).

In the main experiment, RTs to the target grating were excluded from further analysis (a) if the orientation of the target grating was incorrectly reported, (b) if the response was more than 3 standard deviations from that participant’s mean RT within a condition of interest (reflecting lapses or anticipatory responses), and (c) if the size-judgment error on the recall task was 28.5% or more (reflecting a failure to memorize the cued object size). In the size-illusion measurement, difference fractions between the veridical and the reported error size were excluded from further analysis if they were more than 3 standard deviations from the participant average for that particular condition of interest. Data inclusion and participant inclusion are covered in detail in Sections S.2.1 (Experiment 1), S.3.1 (Experiment 2), and S.4.1 (Experiment 3) in the Supplemental Material.

In the main experiment, each size error (*S_E_*) was computed as the unsigned size difference—in percentage—between the reported size (*S_R_*) and the veridical size (*S_V_*), using the equation $S_{E}=100\;\times \;\frac{\left|\;S_{R}-S_{V}\;\right|}{S_{V}}$. Hence, low values (close to zero) reflect small size-recall errors in the memory-recall task, and high values reflect large size-recall errors in the memory-recall task. In the size-illusion measurement, participants’ size judgments (*S_J_*s) were obtained by expressing the reported object size (*S_R_*) as a percentage of the veridical object size (*S_V_*), using the equation $S_{J}\;=100\;\times \;(1+\frac{S_{R}\;-S_{V}}{S_{V}})$. Hence, a percentage above 100% reflects an overestimation of the object size, and a percentage below 100% reflects an underestimation of the object size.

### Data analysis

The relatively large fraction of excluded trials in the main experiments of Experiment 1 (18.8%) and Experiment 2 (13.4%) jeopardized the balancing of observations across experimental conditions. Therefore, we tested our hypotheses using linear mixed-effects models (LMEMs), which circumvent this issue, as they allow for including all individual data points rather than relying on point estimates per condition (Baayen, Davidson, & Bates, 2008; Magezi, 2015).

Because many different LMEMs can be devised for analyzing the same data set, we first compared the potency of an exhaustive range of models (i.e., including all possible combinations of main effects and interaction terms) in describing the observed data. Models were compared using Akaike information criterion (AIC) values, which penalize for the addition of factors (Akaike, 1981; Bozdogan, 1987; see Table S.1). In the Results section, we report statistical tests for the factors included in the best-fitting model to assess whether or not they significantly contributed to describing the observed data. We also report 95% confidence intervals (CIs) for each of these statistical tests; when the interval includes 0, no variance was reliably explained by the factor that was tested. In the Supplemental Material (Sections S.6, S.7, and S.8 for Experiments 1, 2, and 3, respectively), we provide converging evidence from traditional repeated measures analyses of variance (ANOVAs) and Student’s *t* tests (including standardized effect sizes) to facilitate comparison with existing studies.

## Results

### Experiment 1

#### Size-illusion measurement

**Fig. 3.**
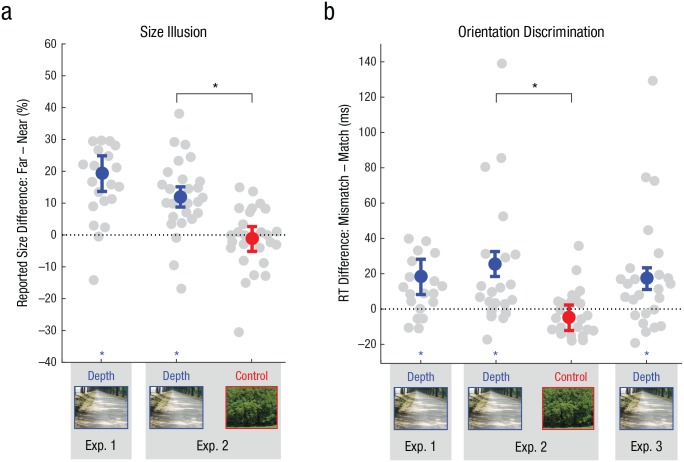
Results of the (a) size-illusion measurement and (b) orientation-discrimination tasks in Experiments 1, 2, and 3. For the size-illusion measurement, positive values depict larger reconstructed object sizes for distant objects than for nearby objects. For the orientation-discrimination tasks, positive values reflect faster response times (RTs) to gratings appearing at the location of template-matching objects than at the location of template-mismatching objects. In both panels, results are depicted for objects presented in depth-inducing scenes (blue) and for objects presented in no-depth control scenes (red). Gray circles represent individual participants, colored circles represent the estimated average effect size from the best-fitting linear mixed-effects model (based on the data of Experiment 1), and error bars represent the 95% confidence intervals for these estimates. Asterisks on the *x*-axis indicate a significant nonzero effect of the factor congruence (i.e., a difference in RTs between gratings appearing at the location of a template-matching and a template-mismatching object), and asterisks above brackets indicate that this effect of congruence significantly differed between conditions (*p* < .005).

First, we aimed to establish whether the scenes and objects used in this experiment induced a size illusion, whereby distant objects were perceived as larger than nearby objects. The LMEM that best described the observed data contained fixed effects for distance and an interaction between distance and shape, along with random effects for object size and participant (see Table S.1). According to this model, distant objects were reported as 19.3% larger (95% CI = [13.7%, 24.9%]) than nearby objects, *t*(1257) = 6.75, *p* < .001. An interaction between distance and shape reflected that this effect was slightly more pronounced for cubes than for spheres, *t*(1257) = 2.06, *p* = .039, 95% CI for the fixed-effect coefficient = [0.2%, 7.6%]. The effect of distance on reported object size (Fig. 3a) was corroborated by a traditional repeated measures ANOVA and was consistently observed across different object sizes, object shapes, and scenes (see Section S.6.1 in the Supplemental Material). Thus, the stimuli employed in Experiment 1 allowed for manipulating the perceived size of physically identical visual objects.

#### Main experiment

Next, we addressed the main research question of whether memory templates favor perceptually matching relative to perceptually mismatching visual objects, even when the competing objects are physically identical. The LMEM that best described the observed data contained fixed effects for congruence and for the interaction between congruence and template size, and a random effect for participant (see Table S.1). According to this best-fitting model, participants were 17 ms faster (95% CI = [6 ms, 27 ms]) at reporting the orientation of a target grating when it was presented at the location of a template-matching visual object (mean from model fit = 528 ms) compared with a template-mismatching visual object (544 ms), as reflected by a significant effect of congruence, *t*(2077) = 3.04, *p* = .002 (see Fig. 3b). A significant interaction between congruence and template size reflected that the congruence effect was slightly larger when participants memorized the larger of the two presented objects, *t*(2077) = 1.99, *p* = .046, 95% CI for the fixed-effect coefficient = [0 ms, 24 ms]. The main effect of congruence was corroborated by a traditional repeated measures ANOVA (see Section S.6.2 in the Supplemental Material). Taken together, these data support the hypothesis that the visual system favors objects that perceptually match the size of a concurrent memory template, even when the competing objects are physically identical.

When reporting the orientation of a target grating, participants were 93.6% accurate (*SD* = 5.4) when the grating appeared at the location of a template-matching object and 94.7% accurate (*SD* = 4.4) when it appeared at the location of a template-mismatching object. Unlike RT, accuracy did not significantly differ between these two conditions, *t*(19) = −1.55, *p* = .137, suggesting that the increase in performance at the location of the template-matching object was RT specific. At the same time, these data provide no evidence for the existence of a speed/accuracy trade-off. Note that *t* tests were conducted on participant means because the trial-based LMEM approach used for the RT data could not be trivially applied to the binary accuracy measure (for comparison, the same analysis approach was applied to the RT data in the Supplemental Material; see Section S.6.2). On average, the reported object size differed 15.1% (*SD* = 3.4) from the actual size of the object that had to be memorized.

### Experiment 2

#### Rationale and prediction

The purpose of Experiment 2 was twofold. First, we aimed to replicate the findings of Experiment 1, basing our sample size on the effect size obtained in Experiment 1 and following the exact same preprocessing and analysis pipeline. Second, we aimed to address an alternative explanation of the pattern of results observed in Experiment 1. In Experiment 1, the perceptually large object in the scene was always presented above fixation, and the perceptually small object was always presented below fixation. Consequently, the results of Experiment 1 could also simply reflect that maintaining a large object in memory biases perception toward the upper visual field, whereas maintaining a small object in memory biases perception toward the lower visual field. To test this possibility, we included an additional condition with scenes that should not induce a size illusion. We expected that the results of Experiment 1 would be replicated when depth-inducing scenes were presented but not when no-depth control scenes were presented.

#### Data analyses

The data from Experiment 2 were analyzed using the LMEMs that best described the data in Experiment 1. By taking this approach, we ensured that the initial model selection was hypothesis free (i.e., data-driven) and that the statistical tests in Experiment 2 were confirmatory rather than exploratory. The present findings were corroborated by performing model comparisons on the data of Experiment 2 (see Sections S.3.2 and S.3.3 in the Supplemental Material).

#### Size-illusion measurement

Before addressing the main research question, we needed to establish that, in contrast to the depth-inducing scenes, the no-depth control scenes did not induce a size illusion. To address this question, we first contemplated only the condition with depth-inducing scenes, using the best-fitting LMEM from Experiment 1. According to this model, distant objects were reported to be 14.6% larger (95% CI = [10.5%, 18.6%]) than nearby objects, as revealed by a main effect of distance, *t*(1637) = 7.07, *p* < .001. An interaction between distance and shape showed that this distance modulation was slightly stronger for cubes than for spheres, *t*(1637) = 2.50, *p* = .013, 95% CI for the fixed-effect coefficient = [0.9%, 7.9%]. These findings replicate those of Experiment 1. In the condition with no-depth control scenes, in contrast, there was no difference between nearby and distant objects, *t*(1633) = −0.31, *p* = .753, 95% CI for the fixed-effect coefficient = [−6.4%, 4.6%]. The interaction between distance and shape was not significant either, *t*(1633) = 0.45, *p* = .654, 95% CI for the fixed-effect coefficient = [3.1%, 5.0%]. Thus, it appears that the scenes that were chosen for the no-depth control condition indeed did not induce a size illusion.

In order to obtain statistical support for this difference between depth-inducing scenes and no-depth control scenes, we ran an aggregate LMEM, which also included fixed effects for depth (depth-inducing or no-depth control scene) and for the interaction between depth and distance. A significant interaction between depth and distance confirmed that the overestimation of distant compared with nearby objects was 13.9% more pronounced (95% CI = [10.1%, 17.7%]) in the depth-inducing condition than in the no-depth control condition, *t*(3271) = 7.16, *p* < .001. The pattern of results obtained here was replicated with LMEM comparisons performed on the data of Experiment 2 (see Section S.3.2 in the Supplemental Material) and with traditional repeated measures ANOVAs (see Section S.7.1 in the Supplemental Material); this pattern was consistent across the full range of object sizes and individual scenes (see Section S.7.1). Taken together, these data confirm that the scenes that were chosen for the depth-inducing and no-depth control conditions were successful in either inducing or not inducing a size illusion, respectively (Fig. 3a).

#### Main experiment

In an attempt to replicate the findings of Experiment 1, we first applied the best-fitting LMEM from Experiment 1 to the condition with depth-inducing scenes from Experiment 2. As in Experiment 1, we found that participants were 24 ms faster (95% CI = [16 ms, 32 ms]) at discriminating gratings that appeared at the location of a template-matching object (mean from model fit = 464 ms) compared with a template-mismatching object (588 ms), as indicated by a main effect of congruence, *t*(2885) = 6.24, *p* < .001. In Experiment 2, this effect did not depend on whether the small or large object was memorized, as revealed by the absence of an interaction between congruence and template size, *t*(2885) = 0.52, *p* = .604, 95% CI for the fixed-effect coefficient = [−6 ms, 11 ms]. Thus, these data replicate the main finding from Experiment 1—that visual objects that match the perceived size of a memory template are favored over mismatching visual objects.

Next, we applied the same LMEM to the condition with no-depth control scenes to investigate whether this effect would persist for scenes that do not induce a size illusion. Here, participants were 6 ms slower (95% CI = [−14 ms, 2 ms]) at discriminating gratings that appeared at the location of a template-matching object (478 ms) compared with a template-mismatching object (472 ms), as indicated by the absence of a main effect of congruence, *t*(2885) = 1.52, *p* = .13. The interaction between congruence and template size did not reach significance either, *t*(2885) = −1.18, *p* = .237, 95% CI for the fixed-effect coefficient = [−14 ms, 3 ms]. In sum, when objects are presented in scenes that do not induce a size illusion, the memory-contingent effect on RTs from Experiment 1 does not replicate.

In order to obtain statistical support for this difference between depth-inducing scenes and no-depth control scenes, we ran an aggregate LMEM, which also included fixed effects for depth and for the interaction between depth and congruence. A significant interaction between congruence and depth revealed that the effect of congruence was 26 ms more pronounced (95% CI = [17 ms, 36 ms]) with depth-inducing scenes than with no-depth control scenes, *t*(5747) = 5.58, *p* < .001. These findings were corroborated by model comparisons of LMEMs based on the data of Experiment 2 (see Section S.3.3 in the Supplemental Material) and traditional repeated measures ANOVAs (see Section S.7.2 in the Supplemental Material). From this, we conclude that the main findings of Experiment 1 are caused by the match between the memory template and the perceived size of the visual objects (as modulated by the scene) and not by a generalized anisotropic deployment of attention following memorization of large or small objects (Fig. 3b).

In the condition with depth-inducing scenes, participants were 94.2% accurate (*SD* = 4.9) when reporting the orientation of a target grating appearing at the location of a template-matching object and 95.0% accurate (*SD* = 5.5) when reporting the orientation of a grating at the location of a template-mismatching object. As in Experiment 1, accuracy did not significantly differ between these two conditions, *t*(25) = −0.89, *p* = .385. Similarly, in the condition with no-depth control scenes, accuracy did not differ between the template-matching (94.1%, *SD* = 5.0) and the template-mismatching (94.5%, *SD* = 5.4) conditions, *t*(25) = −0.55, *p* = .584. Generally, we found no evidence for an accuracy-based increase in performance at the location of the template-matching object and no evidence for a speed/accuracy trade-off. The average recall error in the memory-recall task was 12.2% (*SD* = 2.2).

#### Correlations between the main experiment and size-illusion measurement

Finally, we inquired whether scenes that induce a stronger size illusion would also elicit a stronger template-based attentional-capture effect. To test this, we performed within-subjects correlations between the magnitude of the size illusion (difference in size estimate for nearby and distant objects) and the magnitude of the capture effect (RT difference between gratings presented at template-matching and template-mismatching locations) across each of the 32 scenes. Because of violation of the assumption of normality, Kendall’s τ correlations are reported, and significance at the group level was assessed through bootstrapping (Wilcoxon’s signed-rank test provided similar results).

Across all scenes (16 depth-inducing and 16 no-depth control), those scenes that elicited a stronger size illusion for a participant also elicited a larger attentional-capture effect, average correlation τ = .09 (*SD* = .03), *p* = .001, bootstrapped 95% CI = [.03, .14], 1 × 10^5^ samples. Moreover, this correlation was observed even when we considered only the condition with depth-inducing scenes, average correlation τ = .09 (*SD* = .04), *p* = .009, bootstrapped 95% CI = [.03, .16], 1 × 10^5^ samples (see also Section S.7.3 in the Supplemental Material). Thus, the scenes that induced a stronger size illusion for a particular participant also caused stronger template-based attentional capture for that participant. This suggests that the main finding reported in this manuscript, the template-based attentional-capture effect, genuinely builds on perceived object size as inferred from initial scene analysis.

### Experiment 3

#### Rationale and prediction

Experiment 3 was designed to test whether the allocation of attention toward template-matching objects occurred automatically (i.e., attentional capture) as opposed to volitionally. Because the two objects in the scene were equally uninformative for the upcoming recall task, participants had no objective motivation to volitionally allocate more attentional resources to the template-matching (compared with the template-mismatching) object. We therefore interpreted the findings of Experiments 1 and 2 as reflecting automatic capture of attention by template-matching objects. Nonetheless, we cannot exclude the possibility that participants falsely believed that the (perceptually more similar) template-matching objects were of the same, or similar, size as the objects that should be reproduced during the upcoming memory task. This false belief would incite participants to volitionally attend to the template-matching objects.

In Experiment 3, we tackled this issue by changing only one crucial aspect of the experimental design: After the memorization phase, participants performed either the memory-recall task or the orientation-discrimination task but never both (see Fig. 2). These two trial types were intermixed, thereby requiring participants to memorize the cued-object size on every trial. Crucially, because the orientation-discrimination task was never followed by a recall task, it no longer made sense for participants to attend the template-matching object in aid of the upcoming memory task, as there was no upcoming memory task. Consequently, if we still observed enhanced target-grating discrimination in Experiment 3 at the location of template-matching objects compared with template-mismatching objects, this could not be accounted for by a strategic allocation of attention toward the template-matching objects.

#### Data analyses

Data from Experiment 3 were analyzed using the LMEMs that best described the data in Experiment 1. By doing this, we ensured that initial model selection was hypothesis free (i.e., data driven) and that the statistical tests in Experiment 3 were confirmatory rather than exploratory. The present findings were corroborated by performing model comparisons on the data of Experiment 3 (see Section S.4.2 in the Supplemental Material).

#### Main experiment

In order to replicate the findings of Experiments 1 and 2, we first applied the best-fitting LMEM from Experiment 1 to the data of Experiment 3. As in Experiment 1, we found that participants were 18 ms faster (95% CI = [11 ms, 26 ms]) at discriminating gratings that appeared at the location of a template-matching object (mean from model fit = 477 ms) than at the location of a template-mismatching object (496 ms), as reflected by a main effect of congruence, *t*(2904) = 4.78, *p* < .001. In Experiment 3 (as in Experiment 2), this effect did not depend on whether the small or large object was memorized, as revealed by the absence of an interaction between congruence and template size, *t*(2904) = 1.12, *p* = .265, 95% CI for the fixed-effect coefficient = [−14 ms, 4 ms]. Thus, these data replicate the main finding from Experiments 1 and 2 (Fig. 3b) while precluding strategical biases toward the template-matching objects.

As in Experiments 1 and 2, participants’ accuracy in reporting the orientation of the target grating did not reliably differ between the template-matching condition (92.4% accurate, *SD* = 5.0) and the template-mismatching condition (91.7% accurate, *SD* = 47.2), *t*(24) = 0.65, *p* = .524. Again, we found no evidence for an accuracy-based increase in performance at the location of the template-matching object and no evidence for a speed/accuracy trade-off. On average, participants had a recall error of 11.8% (*SD* = 2.2) in the memory task.

## General Discussion

In naturalistic vision, the behaviorally relevant interpretation of specific visual input (e.g., of a particular object in the world) is dependent on the context within which it is embedded. Here, we investigated whether template-based visual selection, a fundamental property of current theories of visual search, could in principle apply to naturalistic vision given these contextual interactions. Across three experiments, we demonstrated that physically identical visual objects are differently affected by concurrent memory templates when they are presented at different depth planes of a visual scene such that one appears larger than the other. Specifically, when observers memorized a large object, their attention was automatically drawn toward the larger object, compared with a smaller object that produced the same image on the retina; the reverse was true as well. Because the retinal size of the competing objects was identical, template-based selection necessarily operated on the object representations that were rescaled on the basis of the scene context. This implies that scene context modulates object representations before they are compared with a concurrent memory template, thus allowing for template-based selection to accommodate the contextual dependencies that are typical of naturalistic vision.

In addition to providing insight into naturalistic search mechanisms, our findings also contribute to the literature on working-memory-based attentional capture (for a review, see Soto, Hodsoll, Rotshtein, & Humphreys, 2008). To the best of our knowledge, this is the first evidence that size-based working memory templates can induce automatic shifts of attention, thus extending previous observations of color-based and shape-based templates (e.g., Olivers et al., 2006; Soto, Heinke, Humphreys, & Blanco, 2005). Moreover, the current findings are the first to demonstrate that memory templates can bias visual selection toward an object that is perceptually different from—but physically identical to—distractor objects.

Template-based visual selection and scene context are both regarded as important factors underlying naturalistic visual search, yet little is known about how these factors interact (Wolfe & Horowitz, 2017). Focusing on the size dimension, it is known that inferred object distance modulates the size of object representations as early as the primary visual cortex (V1; Fang, Boyaci, Kersten, & Murray, 2008; He, Mo, Wang, & Fang, 2015; Murray, Boyaci, & Kersten, 2006; Ni, Murray, & Horwitz, 2014; Schwarzkopf, Song, & Rees, 2011; for a review, see Sperandio & Chouinard, 2015). There is some debate, however, as to whether such depth-dependent V1 responses are driven by early lateral projections occurring 30 ms to 60 ms after stimulus presentation (Ni et al., 2014) or by later top-down projections occurring around 150 ms after visual stimulation (Chen, Sperandio, Henry, & Goodale, 2019). Either way, the current finding that memory templates discriminate between visual objects that differ only on the basis of their context implies that the interaction between memory templates and visual-object representations takes place later in the visual processing hierarchy than the interaction between scene processing and object processing. Mnemonic and sensory representations of visual objects have been shown to coincide and enhance one another in relatively high-level visual-processing areas, including V4 (Bichot, Rossi, & Desimone, 2005; Chelazzi, Miller, Duncan, & Desimone, 2001), the inferotemporal cortex (Chelazzi, Duncan, Miller, & Desimone, 1998), and lateral occipital and superior parietal areas (Gayet et al., 2017). In light of our current findings, we speculate that an initial gist-based scene analysis modulates early responses to visual objects presented within the scene, the result of which is then fed forward to higher-level visual areas where it is compared with the concurrent memory template.

In the current study, we induced memory templates by instructing participants to memorize an object for subsequent recall rather than by instructing participants to search for a particular object. Earlier research has shown that sustained visual search requires visual memory (Carlisle, Arita, Pardo, & Woodman, 2011; Chun, 2011; Chun, Golomb, & Turk-Browne, 2011; Hodsoll & Humphreys, 2005) and that search instructions and memorization instructions induce memory templates that are qualitatively equivalent (Bundesen, Habekost, & Kyllingsbæk, 2005; Carlisle et al., 2011; de Fockert, Rees, Frith, & Lavie, 2001; Gunseli, Meeter, & Olivers, 2014). A critical difference between the two types of instructions, however, is that while search instructions provide a direct incentive for participants to attend the object that matches the to-be-searched-for feature (e.g., a specific size, as in Hodsoll, Humphreys, & Braithwaite, 2006), a memorization instruction does not. The current findings, which were brought about by a memorization instruction, thus underline the automatic nature of context-dependent template-based visual selection. The automaticity of this effect was confirmed by the findings of Experiment 3, in which the bias toward template-matching objects persisted when strategical shifts of attention were precluded.

The current observation that template-based visual selection can take into account scene-object interactions makes working-memory-based search strategies a viable mechanism for naturalistic visual search. Future research will establish whether the current findings indeed generalize to context-dependent features other than size, such as shape, brightness, and color. Scene–object interactions in template-based visual search could also be accounted for by other mechanisms. For instance, rather than altering the representation of the object that is compared with the template (as shown in the present study), observers could also alter the template before processing the object—for example, by decreasing or increasing the template size when searching for objects at greater or lesser distances, respectively. Whether or not the visual system utilizes this strategy remains a question for future research. Yet another possibility is that observers create memory templates that are distance, illumination, or viewpoint invariant. In line with such a possibility, findings have shown that when observers search for a person among cars (or a car among people), briefly presented person silhouettes capture attention to a similar extent when they are upright or rotated (Reeder & Peelen, 2013), suggesting that the memory template is orientation invariant. Bravo and Farid (2009) showed that, even when not fully invariant, search templates can be resilient to small transformations of size and orientation.

All three mechanisms described here have computational advantages and drawbacks. Considering the efficiency with which we extract information from natural scenes (Bar, 2004; Peelen & Kastner, 2014), these mechanisms might all jointly contribute to effective template-based visual selection in naturalistic visual search.

## Conclusion

The present findings show that human observers can, in principle, use size-based memory templates to favor template-matching visual objects at the expense of template-mismatching visual objects, even when the competing objects produce the same image on the retina. This implies that the representation of visual objects is modulated by the scene context before being compared with current memory templates, thus providing a means for effective template-based visual selection under naturalistic viewing conditions.

## Supplemental Material

Gayet_OpenPracticesDisclosure_rev – Supplemental material for Scenes Modulate Object Processing Before Interacting With Memory Templates Click here for additional data file. Supplemental material, Gayet_OpenPracticesDisclosure_rev for Scenes Modulate Object Processing Before Interacting With Memory Templates by Surya Gayet and Marius V. Peelen in Psychological Science

## Supplemental Material

Gayet_Supplemental_Material_Sections_S1-S4 – Supplemental material for Scenes Modulate Object Processing Before Interacting With Memory Templates Click here for additional data file. Supplemental material, Gayet_Supplemental_Material_Sections_S1-S4 for Scenes Modulate Object Processing Before Interacting With Memory Templates by Surya Gayet and Marius V. Peelen in Psychological Science

## Supplemental Material

Gayet_Supplemental_Material_Sections_S5-S8 – Supplemental material for Scenes Modulate Object Processing Before Interacting With Memory Templates Click here for additional data file. Supplemental material, Gayet_Supplemental_Material_Sections_S5-S8 for Scenes Modulate Object Processing Before Interacting With Memory Templates by Surya Gayet and Marius V. Peelen in Psychological Science
