# Supplementary material for: Scenes Modulate Object Processing Before Interacting With Memory Templates
Source: Psychol Sci. 2019 Sep 16;30(10):1497–509. doi: 10.1177/0956797619869905 (PMC6787763; doi:10.1177/0956797619869905)
Supplement: Gayet_Supplemental_Material_Sections_S1-S4 – Supplemental material for Scenes Modulate Object Processing Before Interacting With Memory Templates [file Gayet_Supplemental_Material_Sections_S1-S4.pdf]

Supplementary materials (SOM-R)

S.1. Overview of experimental factors

Table S.1. *Overview of factor names and factor levels, and whether (+) or not (-) they contributed to describing the observed data in Experiments 1 and 2 (either as main effect or as part of an interaction).*

Factor name	Factor levels	Exp.		
Size illusion measurement		***1***	***2***	***3***
Distance	Object is presented above ('far plane') or below fixation ('near plane')	+	+	
Depth	Depth-inducing or no-depth control scene (Experiment 2 only)		+	
Object Shape	Object is a cube or a sphere	+	+	
Object Size	Object has one of 16 possible sizes	+	+	
Scene	One of 16 possible scenes per Depth condition	-	-	
Order	Depth-inducing or no-depth condition performed first (Experiment 2 only)		-	
Participant	One of N participant (always included as a random effect)			
Main experiment				
Congruence	Grating appears at location of template-matching or mismatching object	+	+	+
Depth	Depth inducing or no-depth control scene (Experiment 2 only)		+	
Template Size	Large or small memory object was memorized	+	-	-
Cue	First or second memory object was memorized	-	-	-
Object Shape	Object is a cube or a sphere	-	-	-
Object Size	Object has one of 16 possible sizes	-	-	-
Scene	One of 16 possible scenes in each Depth condition	-	-	-
Order	Depth-inducing or no-depth condition performed first (Experiment 2 only)		+	
Participant	One of N participant (always included as a random effect)			

Note. An increase or decrease in model fit (based on AIC values) after inclusion of a factor (either as main effects or as part of an interaction), was regarded as evidence for whether (+) or not (-) said factors contributed to describing the observed data in Experiments 1 and 2 (two right-most columns).

S.2. Supplemental results: Experiment 1

S.2.1. Data exclusion

Two participants were excluded from further analyses because they did not perform above chance level (50% correct, binomial test) at reporting the orientation of the target grating (48.4% and 44.5% correct). The 20 included participants were 93.5% accurate (*SD* = 5.0) at reporting the orientation of the target grating. In the memory recall task, participants' average reported object size differed 15.1% (*SD* = 3.4) from the actual size of the object that had to be memorized. All participants performed significantly better than the 28.5% error threshold (one-sided *t*-test) on the memory recall task.

In the main experiment, response times from a total of 2080 trials (out of 2560) were included in the analyses, of which 1038 trials in the template-matching condition, and 1042 in the template-mismatching condition. Trials were excluded for either of three possible reasons: 1.1% (*SD* = 0.9) of response times were not within 3 SDs of participants' mean response times; 6.5% (*SD* = 5.0) of trials yielded an incorrect report of the target orientation; 12.6% (*SD* = 6.9) of trials yielded a size recall error of at least 28.5%.

In the size illusion measurement, 1.0% (*SD* = 0.7) of trials were excluded from further analyses, because perceived object sizes in those trials were not within three SDs of participants' mean perceived object sizes within each condition (arguably reflecting lapses). In total, 1260 of 1280 trials were analyzed in the size illusion measurement (627 trials with objects in the near plane, and 633 in with objects in the 'far' plane).

S.3. Supplemental Results: Experiment 2

S.3.1. Data exclusion

Two participants were excluded from further analyses because they did not perform above chance level (50% correct, binomial test) at reporting the orientation of the target grating (48.4%, and 49.2%). The included participants were 94.2% accurate (*SD* = 4.4). Three participants were excluded for performing at chance level on the memory recall task (recall error of 31.4%, 30.0%, and 28.1% of the to-be-memorized size), according to a one-sided t-test against chance (28.5%). For the included participants, the average recall error was 12.2% (*SD* = 2.2).

In the condition with depth-inducing scenes, response times from a total of 2888 trials (out of 3328) were included in the analyses, of which 1452 trials in the template-matching condition and 1436 in the template-mismatching condition: 1.7% (*SD* = 1.1) of response times were not within 3 SDs of participants' mean response times; 5.8% (*SD* = 4.4) of trials yielded an incorrect report of the target orientation; 6.7% (*SD* = 4.4) of trials yielded a size recall error of at least 28.5%. In the condition with no-depth control scenes, response times from a total of 2863 trials (out of 3328) were included in the analyses, of which 1423 trials in the template-matching condition and 1440 in the template-mismatching condition: 1.4% (*SD* = 0.8) of response times were not within 3 SDs of participants' mean response times; 5.8% (*SD* = 4.8) of trials yielded an incorrect report of the target orientation; 7.4% (*SD* = 4.2) of trials yielded a size recall error of at least 28.5%.

In the size illusion measurement, a total of 3276 trials were analyzed, while 52 trials were excluded from further analyses, because perceived object sizes in those trials were not within three SDs of participants' mean perceived object sizes within each condition. In the condition with depth-inducing scenes 822 'near' trials and 818 'far' trials were included (i.e., an average of 0.9% of excluded

trials per participant; SD = 0.7), and in the condition with no-depth control scenes this included 818 'near' trials and 818 'far' trials (i.e., an average of 1.1% of excluded trials per participant; SD = 1.0).

S.3.2. Model Comparisons: Size Illusion

We performed model comparisons to determine which factors contributed to describing the observed data in Experiment 2 and to assess whether these were the same factors that contributed to describing the observed data of Experiment 1 (see Table S.1.). Model comparisons applied to the full data set of Experiment 2 revealed that the model reported in the main manuscript (i.e., the winning model for the data of Experiment 1, with the additional factors-of-interest Depth and Depth*Distance) was also the model that best described the data of Experiment 2.

Model comparisons applied to only the condition with depth-inducing scenes of Experiment 2 (i.e., an exact replica of Experiment 1) revealed that the exact same factors that best described the data of Experiment 1 also described the data of Experiment 2. Model comparisons applied to only the condition with no-depth control scenes of Experiment 2 revealed that only the fixed factor Object Shape and the random factor Object Size contributed to describing the observed data (thus excluding the factor Distance). These additional model comparisons performed on the data of Experiment 2 thus underline the robustness of the findings reported in the main manuscript.

S.3.3. Model Comparisons: Main Experiment

On the basis of the full data set of Experiment 2, the winning model comprised main effects for, and an interaction term between, the fixed factors Congruence and Depth, akin to the model reported in the Results section of the main manuscript. Contrary to the winning model of Experiment 1, the winning model for describing the data of Experiment 2 did not comprise an interaction term with the factor of non-interest Template Size. According to this winning model, significant effects were observed for Congruence, $t(5747) = 6.69$, $p < 0.001$, 95% CI [15.9ms, 29.0ms], Depth, $t(5746) = 4.18$, $p < 0.001$, 95% CI [7.5ms, 20.6ms], and the interaction between Congruence and Depth, $t(5747) = 5.58$, $p < 0.001$, 95% CI [17.2ms, 35.8ms]. This corroborates the findings obtained with the model reported in the main manuscript.

The model that best described the data of Experiment 2, when considering only the condition with depth-inducing scenes, only comprised a main effect for the fixed factor Congruence, $t(2886) = 7.35$, $p < 0.001$, 95% CI [16.7ms, 28.9ms]. When considering only the condition with no-depth control scenes, the model that best described the data of Experiment 2 only comprised a main effect of the fixed factor Order, $t(2881) = 2.34$, $p = 0.019$, 95% CI [14.8ms, 16.6ms]. Hence, inclusion of the factor Congruence did not contribute to describing the observed data in this condition, mirroring the findings reported in the main manuscript.

S.4. Supplemental Results: Experiment 3

S.4.1. Data exclusion

One participant was excluded from further analyses for performing below chance level (50% correct) at reporting the orientation of the target grating (37.9%). The included participants were 92.0% accurate (*SD* = 5.5). One other participant was excluded for performing at chance level on the memory

recall task (recall error of 25.6% of the to-be-memorized size), according to a one-sided t-test against chance (28.5%). For the included participants, the average recall error was 11.8% (*SD* = 2.2). Finally, a third participant was excluded for having an average response time (838 ms) that was 4.7 *SD*s above the group average (485 ms, *SD* = 73).

In the condition with depth-inducing scenes, response times from a total of 2907 trials (out of 3200) were included in the analyses, of which 1459 trials in the template-matching condition and 1448 in the template-mismatching condition: 1.3% (*SD* = 0.7) of response times were not within 3 SDs of participants' mean response times; 8.2% (*SD* = 5.5) of trials yielded an incorrect report of the target orientation.

S.4.2. Model Comparisons: Main Experiment

On the basis of the full data set of Experiment 3, the winning model comprised only a main effect for the fixed factor Congruence (akin to the condition with depth-inducing scenes of Experiment 2). According to this winning model, there was a significant main effect of Congruence, $t(2905) = 5.07$, $p < 0.001$, 95% CI [9.7ms, 22.0ms]. This corroborates the findings obtained with the model reported in the main manuscript.
