# Supplementary material for: Scenes Modulate Object Processing Before Interacting With Memory Templates
Source: Psychol Sci. 2019 Sep 16;30(10):1497–509. doi: 10.1177/0956797619869905 (PMC6787763; doi:10.1177/0956797619869905)
Supplement: Gayet_Supplemental_Material_Sections_S5-S8 – Supplemental material for Scenes Modulate Object Processing Before Interacting With Memory Templates [file Gayet_Supplemental_Material_Sections_S5-S8.pdf]

Supplementary materials (SOM-U)

S.5. Supplemental methods

S.5.1. Set-up

Stimulus presentation and data collection were performed on a Dell Precision T3610 computer running Windows 7 (64-bit), MatLab 2017a, and the Psychophysics Toolbox 3 (Brainard, 1997; Pelli; 1997), and equipped with a BenQ XL2420T monitor (1920*1080 pixels). Responses were acquired via button presses on a Dell KB212-B keyboard, and read out time was contingent upon the refresh rate of the monitor (144 Hz). A chinrest ensured a fixed viewing distance of 57 cm to the monitor, which was the only source of light in the room during the experiments. All stimuli were presented on top of a gray background with a luminance of 40 Cd/m^2, and a fixation bullseye was presented at the center of the screen throughout the experiment (black outer ring of 0.2 dva, white inner circle of 0.1 dva; 99.6% Michelson contrast).

S.5.2. Additional Stimulus specifications

The horizon (i.e., vanishing point) of each depth-inducing scene was situated within the upper 1 dva of the image, and the original pictures were taken from a normal human viewing height. These images had an average luminance of 40 Cd/m^2 (*SD* = 22), and an average Root Mean Square contrast of 0.21 (*SD* = 0.08). The no-depth control scenes had an average luminance of 47 Cd/m^2 (*SD* = 19), and an average Root Mean Square contrast of 0.24 (*SD* = 0.05).

The target grating had a spatial frequency of 7 cycles per degree and a peak Michelson contrast of 0.5, and a circular envelope with a total diameter of 0.8 degrees, which included a cosine-shaped contrast decrement along the edge (width: 0.3 dva).

The visual objects presented in the scenes had an average size of 0.7*0.7 dva, an average luminance of 35 Cd/m^2 (*SD* = 17), and an average Root Mean Square contrast of 0.21 (*SD* = 0.07).

S.6. Supplemental Results: Experiment 1

S.6.1. Size Illusion (Traditional Analyses)

A planned pairwise *t*-test showed that objects were reported to be (15.7%, *SD* = 11.8) larger when they were presented in the far plane (122.5% of the veridical object size, *SD* = 12.0) compared to the near plane of a scene (106.8%, *SD* = 10.3), $t(19) = 5.96$, $p < 0.001$, Cohen's $d = 1.33$. This suggests that the stimuli employed in Experiment 1 allowed us to manipulate the perceived size of physically identical visual objects.

The size overestimation of visual objects presented on the far plane relative to the near plane was consistently observed across the different object sizes (Figure S.1, Panel A, left), scenes (Figure S.1, Panel A, middle), and object shapes (cubes or spheres; Figure S.1, Panel A, right) included in this experiment.

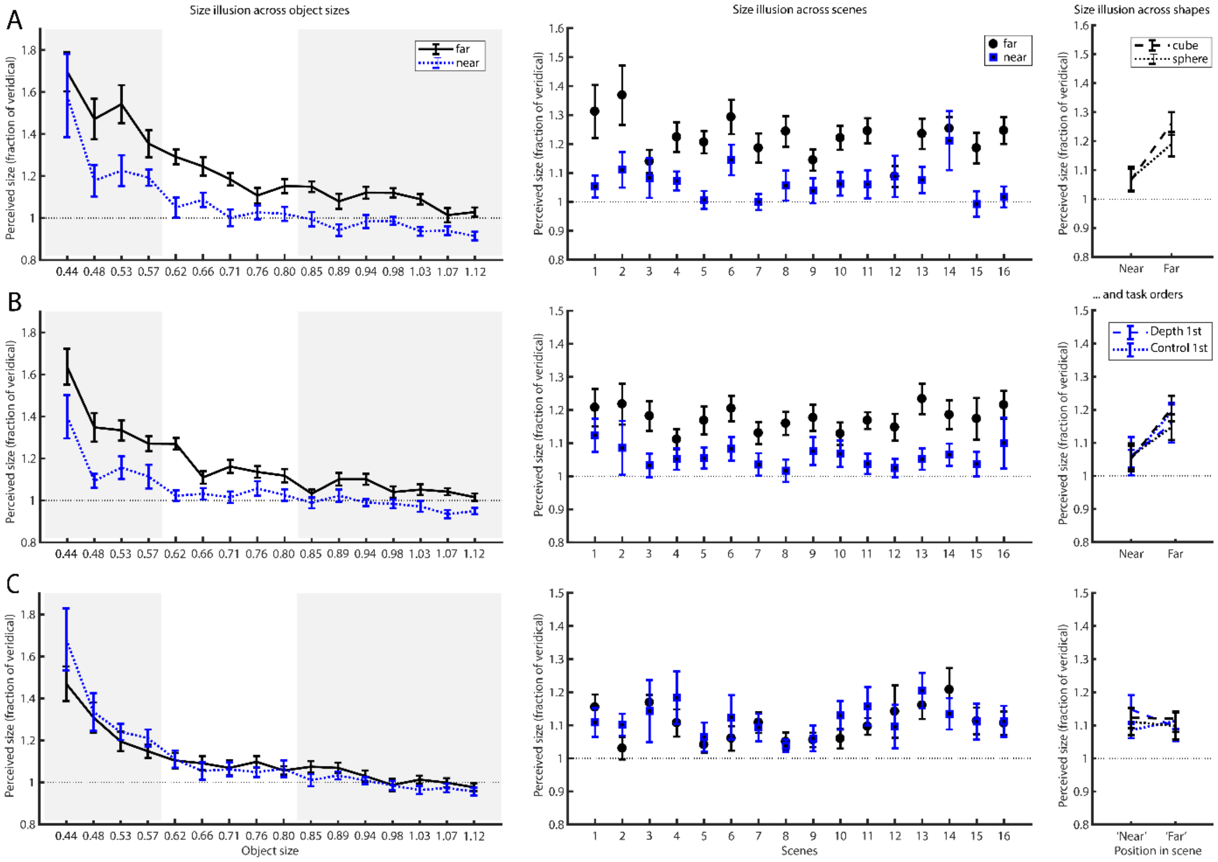

Figure S.1. Size illusion across object sizes (left), scenes (middle) and object shapes (right), for Experiment 1 (Panel A), and Experiment 2 (Panel B and C for the depth-inducing condition, and the no-depth control condition respectively). The magnitude of the size illusion (on the y-axis) is computed by dividing the reported size by the veridical size of the visual object. The shaded area in the left-most graphs reflect sizes that were used as memory items, but not as probe items in the main experiment. Error bars in the left-most and middle graphs depict the standard error of the mean. Error bars in the right-most graph reflect the 95% confidence intervals of the within-subject difference between the 'near' and 'far' objects (Morey, 2008).

S.6.2. Main Experiment (Traditional Analyses)

A 2x2 repeated-measures ANOVA with the factors Template Size and Congruence yielded no interaction effect between Template Size and Congruence ($p > 0.3$, $\eta^2 < 0.1$), no main effect of Template Size ($p > 0.1$, $\eta^2 < 0.1$), but a significant main effect of Congruence, $F(1, 19) = 7.59$, $p = 0.013$, $\eta^2 = 0.29$. After collapsing across Template Size conditions, the planned pairwise *t*-test revealed that observers were indeed 11ms (*SD* = 16) faster at reporting the orientation of a target grating when it was presented at the location of a template-matching visual object (527ms, *SD* = 80) compared to a template-mismatching visual object (538ms, *SD* = 84), $t(19) = 2.85$, $p = 0.010$, *Cohen's d* = 0.64. This result supports the hypothesis that the visual system favors objects that perceptually match (rather than mismatch) the size of a concurrent memory template, even when the competing objects are physically identical.

S.7. Supplemental Results: Experiment 2

S.7.1. Size Illusion (Traditional Analyses)

A repeated-measures ANOVA was conducted to investigate whether the within-subject factors Distance (i.e., object presented in the top or bottom of the scene), and Depth (scenes induced depth or not), influenced the reported size of the visual objects presented within the scenes. This analysis revealed a main effect of Distance, $F(1, 25) = 11.95$, $p = 0.002$, $\eta^2 = 0.32$, and – most importantly – the expected interaction between Distance and Depth, $F(1, 25) = 21.20$, $p < 0.001$, $\eta^2 = 0.46$, but no main effect of Depth, $F(1, 25) = 0.21$, $p = 0.651$, $\eta^2 = 0.01$.

To assess whether the interaction between Distance and Depth reflected a size illusion in depth-inducing scenes, but not in no-depth control scenes, subsequent planned *t*-tests were conducted. Paired-samples *t*-tests confirmed that objects were reported as 12.0% (*SD* = 11.7) larger when presented in the far plane (117.6% of the veridical object size, *SD* = 11.5) compared to the near plane (105.6%, *SD* = 11.2) of depth-inducing scenes, $t(25) = 5.23$, $p < 0.001$, Cohen's d = 1.03. In no-depth control scenes, however, size judgements were comparable (i.e., -1.2% difference, *SD* = 9.8) between the 'far plane' (110.4%, *SD* = 7.6) – or top of the scene – and 'near plane' (111.6%, *SD* = 12.8) – or bottom of the scene, $t(25) = -0.62$, $p = 0.54$, Cohen's $d = -0.12$, $BF_{0+} = 7.3$.

This overestimation of the size of visual objects on the far plane relative to the near plane in depth-inducing scenes was consistently observed across the different object sizes (Figure S1, Panel B, left), scenes (Figure S1, Panel B, middle), and object shapes (cubes or spheres; Figure S1, Panel B, right) included in this experiment. The absence of such an overestimation in no-depth control scenes was also consistent across object sizes, scenes, and object shapes (Figure S.1, Panel C).

S.7.2. Main Experiment (Traditional Analyses)

In order to investigate whether the response time benefit for targets appearing at the location of template-matching visual objects would replicate depending on the presented scene type, we conducted a repeated-measures ANOVA with the factors Congruence and Depth. This analysis revealed no significant main effects of Congruence, $F(1, 25) = 4.08$, $p = 0.054$, $\eta^2 = 0.14$, or Depth, $F(1, 25) < 0.01$, $p = 0.996$, $\eta^2 < 0.01$, but did reveal the crucial interaction between Congruence and Depth, $F(1, 25) = 6.01$, $p = 0.022$, $\eta^2 = 0.19$.

Subsequent planned *t*-tests confirmed the nature of this interaction. In the condition with depth-inducing scenes, observers were 24ms (*SD* = 53) faster at reporting the orientation of a target grating when it was presented at the location of template-matching visual object (464ms, *SD* = 97) compared to a template-mismatching visual objects (488ms, *SD* = 109), $t(25) = 2.31$, $p = 0.029$, Cohen's $d = 0.45$. In the condition with scenes that did not induce a size illusion, however, observers were equally fast (i.e., -3ms difference, *SD* = 13) at reporting the orientation of a target grating, when it was presented at the location of a 'template-matching' visual object (477ms, *SD* = 109) compared to a 'template-mismatching' visual object (488ms, *SD* = 109), $t(25) = 1.27$, $p = 0.217$, Cohen's $d = 0.45$, $BF_{0+} = 10.0$.

S.7.3. Within-participant correlation between size illusion and main experiment

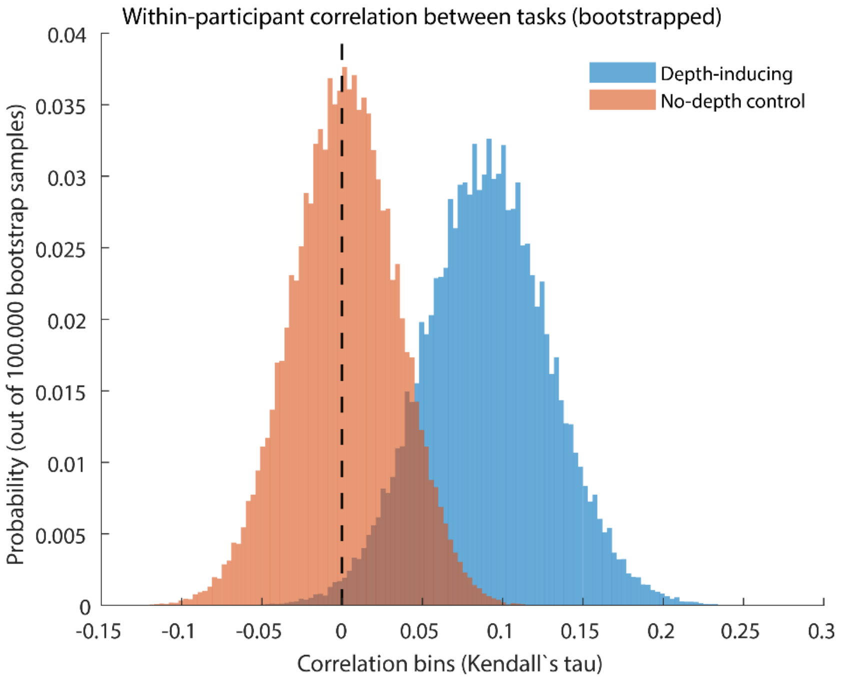

Figure S.2. Average within-participant correlation between the magnitude of the size illusion (near minus far) and the template-contingent capture in the main experiment across the 16 depth-inducing scenes (in blue) and the 16 no-depth control scenes (in red) of Experiment 2.

S.8. Supplemental Results: Experiment 3

S.8.1. Main Experiment (Traditional Analyses)

A 2x2 repeated-measures ANOVA with the factors Template Size and Congruence yielded no interaction effect between Template Size and Congruence ($p > 0.8$, $\eta^2 < 0.01$), no main effect of Template Size ($p > 0.1$, $\eta^2 < 0.1$), but a significant main effect of Congruence, $F(1, 24) = 6.32$, $p = 0.019$, $\eta^2 = 0.21$. After collapsing across Template Size conditions, the planned pairwise *t*-test revealed that observers were indeed 16ms (*SD* = 32) faster at reporting the orientation of a target grating when it was presented at the location of a template-matching visual object (477ms, *SD* = 78) compared to a template-mismatching visual object (493ms, *SD* = 71), $t(24) = 2.53$, $p = 0.018$, *Cohen's d* = 0.51. This result replicates the findings of Experiments 1 and 2, while precluding the possibility of a strategically instantiated bias toward memory-matching stimuli (thus substantiating the automaticity of said bias).
